# Assessing the Variability of Energy Metabolisability in Barley, Rye, and Wheat Varieties for Broiler Diets

**DOI:** 10.3390/ani14243559

**Published:** 2024-12-10

**Authors:** Ibtissam Kaikat, David Solà-Oriol, José Francisco Pérez

**Affiliations:** Animal Nutrition and Welfare Service (SNiBA), Department of Animal and Food Science, Universitat Autònoma de Barcelona (UAB), 08193 Bellaterra, Spain; david.sola@uab.cat (D.S.-O.); josefrancisco.perez@uab.cat (J.F.P.)

**Keywords:** metabolisable energy, cereal variability, nutrient flow, broiler

## Abstract

This trial assessed the variability of energy metabolisability in four varieties of barley, rye, and wheat based on changes in energy and nutrient flow estimations in excreta. Twelve diets were created by combining 40% of each cereal variety and 60% of a common mixture, divided into enzyme-supplemented and non-supplemented versions, resulting in 24 experimental diets that included TiO_2_ and Yb_2_O_3_ as indigestible markers. A total of 432 one-day-old broilers were distributed into 72 cages and fed a single diet from day 1 to 15. Experimental runs were conducted from day 16 to 25 using a crossover design. Diets were alternated between enzyme-supplemented and non-supplemented for each cage. Excreta samples were collected on days 20 and 25. Energy metabolisability was highest (*p* < 0.001) in wheat (83.3%), followed by barley (77.8%) and lowest in rye (70.6%). The variety influenced energy metabolisability in barley and wheat (*p* < 0.001) at a wider range than predicted by NIR analyses. The variety influenced the increment of energy in response to enzyme supplementation in barley diets (*p* < 0.05), with varieties having low metabolisability values showing higher responses. TiO_2_ and Yb_2_O_3_ did not differ in estimating nutrient flow. This study quantifies energy variability among and within cereals and identifies potential variation factors.

## Simple Summary

This study aimed to evaluate the variability of metabolisable energy across different barley, rye, and wheat varieties in broiler diets, with a focus on improving the precision of feed formulation. The metabolisability of energy can vary significantly due to factors like chemical composition and antinutritional contents, making accurate predictions challenging using conventional methods such as feed tables and near-infrared reflectance technology calibrations. The study tested whether a standard ingredient substitution method could determine the nutritional variability of various ingredients in a single trial, comparing the effects of different cereal varieties on the flow of nutrients in excreta and examining the impact of enzyme supplementation. Additionally, the research explored the use of ytterbium oxide as an alternative digestibility marker to titanium dioxide, given concerns over its safety and regulatory status. This trial is expected to enhance the understanding of intrinsic differences in feed ingredients’ metabolisable energy and help improve feed formulation accuracy.

## 1. Introduction

Feed formulation requires a precise definition of the nutritional value of feed ingredients, including energy, amino acids, and P as main references. The basis is that the ingredients (mainly cereals and protein concentrates) are mixed to provide nutritional values in the feed that can be calculated based on their additive contribution. The competitive entry of ingredients into the best feed formulation linear programmes depends on their differential feed value and cost, while the precision of the final feed value will also depend on the digestibility or metabolisability variability of different batches, associated with their chemical composition, antinutritional factor contents, or feed technology processing conditions.

In practice, nutrient values can be acquired using feed tables or estimated using equations, including those included in near-infrared reflectance technology (NIR) calibrations. However, in all these approaches, the prediction of digestibility and metabolisability is usually poor and depends on experimental conditions used for tabulation and calibration [1,2]. In current animal feeding practises, digestibility also depends on relevant animal factors (i.e., type of bird, age, genotype) and environmental farm conditions (i.e., sanitary status, heat stress). It could be argued that there are no unique nutritional values for ingredients, while it raises interest in better quantifying intrinsic differences in feed value among ingredients and their batches as a major driver for precise feed formulation.

This study focused on assessing the energy metabolisability variability among cereals (three cereal species: barley, rye, and wheat, each with four varieties) for broiler chickens. It examined the hypothesis that variability in energy metabolisability values among different cereal species and varieties could be determined in a single trial using a standard ingredient substitution method by focusing on the changes promoted by these diets in the excreta flows. The comparative values will allow a ranking of the ingredients to be established, based on metabolisable values, and to explore sources of variation, including the use of in-feed enzymes (phytase, xylanase, and β-glucanase), which may also help to optimise prediction equations. On the other hand, titanium dioxide (TiO_2_) has historically been the preferred inert marker in digestibility studies due to its high recovery rates and accuracy [3]; however, the concerns about its safety and regulatory status [4] may necessitate the exploration of alternative markers. This study also aims to assess the use of ytterbium oxide (Yb_2_O_3_) as a viable alternative.

## 2. Materials and Methods

### 2.1. Bird Management, Husbandry, Experimental Design, and Diets

In total, 432 one-day-old broilers (Ross 308) were obtained from a local hatchery, where they received *in ovo* vaccinations for Marek disease, Gumboro disease, and infectious bronchitis. The birds were weighed and allotted 72 battery brooder cages with 6 birds per cage. The brooder temperature was maintained at 35 °C from d 0 to d 4 post-hatch and was progressively reduced to 25 °C on d 14. The light cycle was 24 h/d from d 1 to d 2, 23 h/d from d 3 to d 10, and 18 h/day from d 11 to d 25. A single corn, wheat, and extruded soybean-based diet supplemented with feed enzymes was offered from d 1 to d 15 (Table 1). Four different varieties of barley sourced from both Germany and France: *KWS Borrelly* (B1), *KWS Faro* (B2), *KWS Thalis* (B3), and *KWS Tardis* (B4); rye from Germany: *Conduct* (R1), *KWS Serafino* (R2), *KWS Igor* (R3), and *KWS Gilmor* (R4); and wheat from France: *KWS Ultim* (W1), *Solehio* (W2), *KWS Extrem* (W3), and *KWS Sphere* (W4), were chosen to exhibit varying levels of crude protein (CP), starch, and neutral detergent fibre contents and provided by KWS LOCHOW GMBH (Bergen, Germany). Upon reception, the 12 ingredients were ground through a 3 mm screen, and 12 diets were prepared precisely to contain 40% of each cereal variety and 60% of a common basal mixture to satisfy the growing broilers’ requirements (Table 1). Each diet was prepared without and with supplementation of an *in-feed* dose of phytase (1000 FTU/kg), xylanase (16,000 BXU/kg), and β-glucanase (20,000 BU/kg), creating 24 different diets; all enzymes were sourced from AB Vista (Marlborough, UK). All diets included 2 g/kg of TiO_2_ and 50 mg/kg of Yb_2_O_3_ as inert markers. On d 16, the 24 experimental diets were distributed among cages in 2 subsequent experimental runs: from d 16 to d 20 and from d 21 to d 25, with each diet being tested in a crossover design with six replicates (three replicates per run). Each experimental run consisted of 36 cages receiving enzyme-supplemented diets and 36 receiving non-supplemented diets. For each cereal variety, three cages were randomly assigned the supplemented diet and three the non-supplemented diet in the first run. In the second run, cages previously assigned to the non-supplemented diet were switched to the supplemented diet for the same cereal variety, and vice versa. This balanced crossover design ensured that any potential residual effects were equally distributed between treatments, thereby eliminating carry-over effects. All diets met or exceeded the nutrient requirements for broilers [5] and were fed in mash form. Feed and water were provided for ad libitum consumption throughout the experiment.

### 2.2. Excreta Sampling

Excreta were collected over a continuous 24 h period on days 20 and 25 of the experiment. Clean trays were placed under each cage 24 h prior to sampling, and residual feathers or feed were diligently removed before thoroughly mixing the accumulated excreta to ensure homogeneity. Representative samples were then collected, oven-dried at 60 °C for 48 h, and ground through a 0.5 mm screen for further analysis.

### 2.3. Laboratory Analyses

The contents of moisture and CP were analysed in tested cereals, experimental diets, and excreta samples based on AOAC International [6] analytical methods 930.15 and 990.03, respectively. Concentrations of Ti and Yb were determined in diets and excreta samples according to the AOAC 984.27 method using an optical emission spectrometer ICP-OES 5900 (Agilent Technologies, Santa Clara, California, USA). Cereals and excreta samples were analysed for gross energy (GE) according to the UNE-EN ISO 9831-2004 [7] standard using a calorimetric Parr 6300 Calorimeter bomb (Parr Instrument Company, Moline, IL, USA), for ether extract (EE) following the AOAC 945.16 method, and for ash content using the AOAC 942.05 method. Total starch was determined in excreta samples following the AOAC 920.40 method. Cereal samples were analysed for soluble and insoluble non-starch polysaccharides (NSP) following the procedure of Englyst et al. [8] and for rapidly and slowly digestible, resistant, and total starch according to Englyst et al. [9]. Diets were analysed for phytase, xylanase, and β-glucanase activity through an ELISA method using Quantiplate Kits (Enzyme Services & Consultancy, Innovation & Technology Centre, Ystrad Mynach, UK). After undergoing an in vitro simulation of gastric digestion, the cereal samples were left in a test tube at room temperature for 3 h; the swelling capacity (SC) was then determined as the ratio of the liquid to solid phase [10]. The tubes were then centrifuged at 2500× *g* for 10 min; the supernatant was removed; the tubes were inverted and left to drain for 25 min, and then weighed. Water retained by the sample was determined as the weight lost after drying at 103 °C for 16 h; water retention capacity (WRC) was calculated as the percentage of water retained per gram of dry residue [11].

### 2.4. Calculations

Dry matter (DM) flow (per kg DM diet intake) in the excreta was calculated according to the following equation:

(1) $$DM\;flow=M_{diet}/M_{excreta}$$

where M_diet_ and M_excreta_ are the concentrations of Ti or Yb in diets and excreta (DM basis), respectively. The flows of GE, starch, N, and EE (kcal or g/kg DM diet intake) were then calculated as

(2) $$item\;flow={item}_{excreta}\times DM\;flow\;$$

where item_excreta_ is the content of GE, starch, N, or EE (kcal or g/kg DM) in the excreta.

The following equation was developed to estimate the digestibility of a test ingredient in reference to a reference ingredient:

(3) $${item\;flow}_{DT}-{item\;flow}_{DR}={level}_{T}\times \left[{item}_{T}\times \left(1-{digestibility}_{T}\right)-{item}_{R}\times \left(1-{digestibility}_{R}\right)\right]$$

where item flow_DT_ and item flow_DR_ are the flows of the nutritional item (kcal or g/kg, as-fed basis) of the diets containing the test and the reference ingredient, respectively, level_T_ is the inclusion level of test and reference ingredients in the diets; item_T_ and item_R_ are the contents of the nutritional item (kcal or g/kg, as-fed basis) in the test and the reference ingredient, respectively; digestibility_T_ and digestibility_R_ are the digestibility coefficients of the nutritional item in the test and the reference ingredient, respectively. Following this equation, the metabolisability of GE of each cereal was thus calculated as

(4) $${metabolisability}_{T}=1-\left[{(GE\;flow}_{DT}-{GE\;flow}_{DR})/\left(0.4\times {GE}_{T}\right)\right]-\left[\left({GE}_{R}/{GE}_{T}\right)\times \left(1-{metabolisability}_{R}\right)\right]$$

where metabolisability_R_ = 80.4% as the NIR-estimated metabolisability of GE value of W2 (Evonik Operations GmbH, Essen, Germany), which was chosen randomly as the reference ingredient. The comparison and ranking of the test ingredients remain consistent regardless of which ingredient is chosen as the reference. Each test cereal was individually compared to the fixed reference cereal (W2), with calculations accounting for the differences in nutrient and energy flows in the excreta. The variability observed in metabolisability values is attributable solely to the cereal.

The nitrogen-corrected apparent metabolisable energy (AMEn) and standardised apparent metabolisable energy (AME_s_) values (kcal/kg) of each cereal were calculated as

(5) $${AME}_{n\;or\;s}={metabolisability}_{n\;or\;s}\times {GE}_{T}$$

where metabolisability_n or s_ is the calculated energy metabolisability using Equation (4) with GE flow_DT_ and GE flow_DR_ corrected for a zero N balance (GE flow + 8.22 kcal/g N gain) or standardised for 50% retention of dietary N (GE flow + 8.22 × (N gain—0.50 × N intake)) [12,13].

The increment of AME (ΔAME) in response to enzyme supplementation of the diets was calculated as

(6) $$∆AME={GE\;flow}_{D-}-{GE\;flow}_{D+}$$

where GE flow_D−_ and GE flow_D+_ are the flows of GE in excreta, corrected for a zero N balance of the diet without and with enzyme supplementation, respectively.

### 2.5. Statistical Analyses

Data were statistically analysed using an R3.6.3 (The R Foundation for Statistical Computing) user-friendly interface implemented in InfoStat [14]. Two distinct statistical evaluations were conducted. Initially, the impact of cereal species, enzyme supplementation, and marker type on dietary flows in the excreta was examined. Subsequently, the influence of variety, enzyme supplementation, and marker type within each cereal species on dietary flows in the excreta was investigated. To analyse the data, generalised linear mixed models (GLMM) were employed, utilising the following statistical models:

$$Y_{ijklm}=\mu +C_{i}+E_{j}+M_{k}+\left(C_{i}\times E_{j}\right)+\left({pen}_{l}+{run}_{m}\right)+\varepsilon_{ijklm}$$

and

$$Y_{jklmn}=\mu +V_{n}+E_{j}+M_{k}+\left(V_{n}\times E_{j}\right)+\left({pen}_{l}+{run}_{m}\right)+\varepsilon_{jklmn}$$

where Y_ijklm_ and Y_jklmn_ are the dependent traits, μ is the overall mean of the model, C_i_ is the fixed effect of cereal species i (barley, rye, or wheat), E_j_ is the fixed effect of enzyme supplementation j (without or with enzymes), M_k_ is the fixed effect of the marker k (TiO_2_ or Yb_2_O_3_), (C_i_ × E_j_) represents the interaction effect between the cereal and enzyme supplementation, V_n_ is the fixed effect of variety n (B1, B2, B3, or B4; R1, R2, R3, or R4; and W1, W2, W3, or W4), (V_n_ × E_j_) represents the interaction effect between the variety and enzyme supplementation, pen_l_ + run_m_ are cross-random effects, and e_ijklm_ and e_jklmn_ are residual errors. Other interactions were not included because they were not significant. The metabolisability of energy, AME_n_, AME_s_, and ΔAME were analysed using the same two GLMM to examine differences among cereal species and varieties. To provide energy metabolisability values for the ingredients, the models excluded the enzyme supplementation factor and included only data from the non-supplemented diets. Effects were considered to be significant when *p* < 0.05. The associations between in vivo obtained parameters, chemical composition, and NIR-predicted values were explored using Pearson’s correlation analysis and presented using the ‘corrplot’ package in R version 3.6.3.

## 3. Results

### 3.1. Cereal and Diet Analyses

The concentration of nutrients in cereal samples showed clear variations among varieties (Table 2 and Table 3). Higher variability was observed for starch in rye (from 52.8 to 58%) than in wheat (from 57.2 to 61.9%) and barley (from 51.3 to 54.9%); and for CP in wheat (from 9.6 to 13.7%) than rye (from 6.2 to 8.6%) and barley (from 8.1 to 9.9%). Total NSP concentration varied from 10.1 to 15.4% in barley, from 6.8 to 11.9% in wheat, and from 11.1 to 12.5% in rye. In terms of physicochemical characteristics, the average SC values were 456%, 294%, and 179% for rye, barley, and wheat, respectively. Average WRC values were also higher for rye (124%) and barley (105%) compared to wheat (79%).

The enzyme activity analysed in the diets mirrored the planned values, thus meeting the objectives of this trial (Table 4). The level of enzyme in non-supplemented diets likely represents endogenous β-glucanase activity, showing lower values for barley (from 6720 to 10,800 BU/kg) and wheat (from 7420 to 9120 BU/kg) than rye (from 10,600 to 17,600 BU/kg). However, phytase and xylanase activity were not detectable in any of the non-supplemented diets (<50 FTU/kg and <2000 BXU/kg, respectively).

### 3.2. Energy and Nutrient Flow in Excreta

No significant interaction was detected between cereal species and enzyme supplementation on the flow of GE, starch, N, and EE in excreta (Table 5). The flow of GE was highest (*p* < 0.001) in the rye-based diet, followed by the barley-based diet, and lowest in the diet containing wheat. The flow of starch was highest (*p* < 0.001) in the rye-based diet, followed by the wheat-based diet, and lowest in the diet containing barley. No difference in N excretion in excreta was detected among diets, whereas the flow of EE was greater (*p* < 0.05) in the rye-based diet. Broilers fed diets with exogenous enzymes had lower (*p* < 0.001) excretion of GE (–69 kcal/kg), starch (–1.25 g/ kg), N (–0.63 g/kg), and EE (–2.33 g/kg).

Interactions between the variety of barley and enzyme supplementation were significant for GE and N (*p* < 0.001) and starch (*p* < 0.05) excretions in the barley-based diet (Table 6). Enzyme supplementation decreased those excretions in B1 and B4; meanwhile, it had no significant effect on B2 and B3. Enzyme supplementation also decreased the flows of GE and N (*p* < 0.001), as well as starch and EE (*p* < 0.05) in the rye-based diet. No difference was detected among rye varieties. The flows of GE and starch were influenced by both wheat variety (*p* < 0.05) and enzyme supplementation (*p* < 0.05); meanwhile, the flows of N and EE decreased with enzyme supplementation in the wheat-based diet (*p* < 0.001).

**Figure 1:**
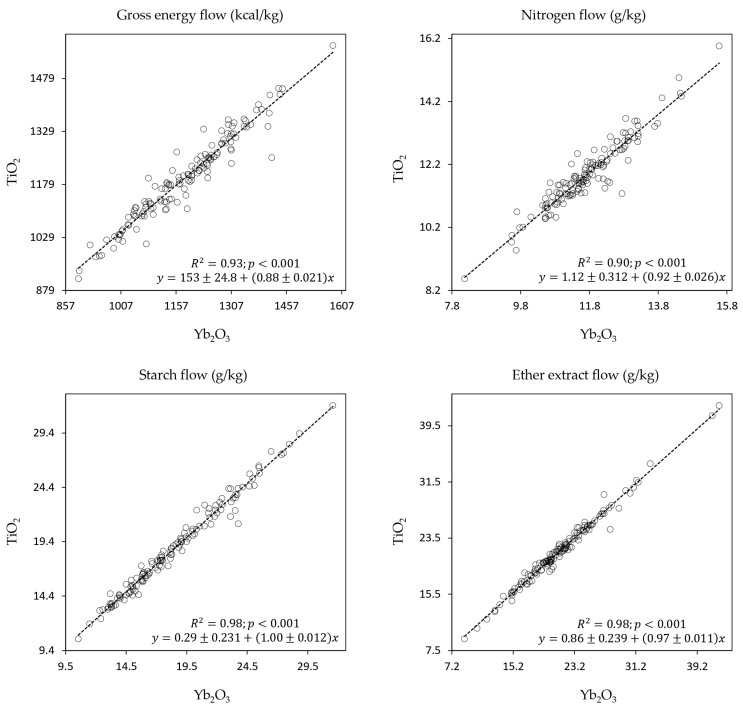
Regression analyses between nutrient flows in excreta (kcal or g per kg diet dry matter) using titanium dioxide (TiO_2_) and ytterbium oxide (Yb_2_O_3_) as indigestible markers in growing broilers.

No significant interactions were observed between the marker type and the cereal species, cereal variety, or enzyme supplementation on GE, starch, N, and EE flows in excreta. No significant differences (*p* > 0.05) were detected between the two indigestible markers in estimating these flows. Additionally, according to the regression analysis between flow estimates using the two types of markers, Yb seems to have similar accuracy as Ti for estimating the variability of GE, starch, N, and EE flows in excreta (Figure 1).

### 3.3. Metabolisability of Energy

The metabolisability of energy was highest (*p* < 0.001) in wheat (83.3%), followed by barley (77.8%) and lowest in rye (70.6%) (Table 7). Similarly, the AME_s_ value was greatest (*p* < 0.001) in wheat (3177 kcal/kg), followed by barley (2991 kcal/kg), and lowest in rye (2665 kcal/kg). The cereal variety influenced energy metabolisability in barley (*p* < 0.001) and wheat (*p* < 0.05), with values ranging from 74.3% for B4 to 83.1% for B3 and from 78.8% for W1 to 86.5% for W4. No differences were observed among varieties in rye. In parallel, differences in AME_s_ between varieties were observed in barley (*p* < 0.001) and wheat (*p* < 0.05), with values ranging from 2854 kcal/kg for B4 to 3181 kcal/kg for B3 and from 2981 kcal/kg for W1 to 3351 kcal/kg for W3. No differences in AME_s_ were observed among varieties in rye. Comparing all the 12 varieties of the 3 cereal species, the energy metabolisability and AME_s_ of B3 (83.1%, 3181 kcal/kg) were not different from wheat varieties; W1 (78.8%, 2981 kcal/kg) was not different from barley varieties; and B1 (74.5%, 2883 kcal/kg) and B4 (74.3%, 2854 kcal/kg) were not different from rye varieties.

The ΔAME tended to be greater (*p* < 0.10) in the rye-based diet than in the barley- and wheat-based diets. The genotype variety influenced ΔAME in barley (*p* < 0.05), with varieties having low metabolisability showing higher responses. No differences in ΔAME were observed between varieties in rye and wheat.

## 4. Discussion

### 4.1. Critical Evaluation of the Method

Various bioassay protocols are employed to determine the nutritional value of ingredients, which are crucial for designing table values, predictive equations, or NIR calibrations. These protocols differ in terms of how the test ingredient is incorporated into diets for animal consumption. Different approaches, as described by Wu et al. [2], include substituting the standard ingredient in a basal diet with the test ingredient (standard ingredient substitution), mixing the test ingredient with a basal diet to create a test diet (basal diet substitution), or blending multiple test ingredients at various independent levels into different test diets (multiple basal substitutions).

The nutritional values measured using these methods can be significantly influenced by several factors, including the health status and physiological conditions of the birds, such as age, sex, breed, and metabolic responses, leading to variability in results. Controlled feeding regimes or variable feed intake due to imbalanced nutrient intake can result in inconsistent endogenous energy losses, affecting the accuracy of metabolisable energy values. Additionally, the assumption that the energy provided by all ingredients is additive may not hold true, as nutrient interactions, such as the extra caloric effect of fat and the interaction between fat and NSP in grains, can influence the overall energy value of the diet. Moreover, the varying ratios of inclusion among basal ingredients, such as minerals, can also contribute to nutritional disparities between the basal and test diets, impacting the evaluation of the test ingredient [2].

Considering these circumstances, this study proposes adopting the standard ingredient substitution method, focusing on replacing the cereal component at a fixed level, which accounts for 40%. This approach aims to maintain the nutritional value of the final test diets as stable as possible while ensuring the levels and consistency of the remaining basal ingredients, which constitute 60% of the diet (including minerals, vitamins, and protein concentrates). Consequently, any differences observed in nutrient flows at the digestive tract or excreta level among test diets can be attributed to the 40% replacement, thereby allowing the estimation of the nutritive values of test ingredients. The significant differences observed in the excreta flows among cereals, varieties, and enzyme supplementation confirm that the inclusion level (40%) of the test ingredient was high enough to allow a good ingredient assessment. Importantly, these differences in nutrient flows are independent of basal endogenous excretions, making them informative indicators of standardised nutritional differences among ingredients.

A potential criticism of this protocol is the assumption of a known energy value for the reference ingredient, whether obtained through NIR estimation or using table values. However, fixing the value of one ingredient as a reference allows us to calculate the relative values of other ingredients. This method highlights the true differences observed in vivo and provides a clear ranking of ingredients. While the reference value itself may not be entirely accurate, it is the differences that are important for feed formulation, as exact values are often difficult to obtain. Furthermore, by focusing on quantifying variations in digestibility values among cereal species and varieties, we can identify compositional factors contributing to these differences, such as starch, CP, and NSP contents, and various physicochemical properties.

### 4.2. Variability of Energy Value Among Cereals and Varieties

The analysed composition of barley, rye, and wheat in this study aligns with values reported in major feed tables (Appendix A Table A1). For instance, the range of CP values observed for barley (8.1–9.9%) is comparable to those reported in various feed databases (8.5–11.7%), while starch content (51.3–54.9%) also falls within the expected range (50.6–53.7%). Similarly, for rye, our CP values (6.2–8.6%) and starch values (52.8–58%) are consistent with reported ranges of 8.5–12.1% for CP and 53.7–59.6% for starch. Wheat demonstrated comparable ranges as well, with CP values (9.6–13.7%) and starch content (57.2–61.9%) aligning closely with the reported ranges of 10.2–12.9% for CP and 56.7–61.8% for starch.

AME_n_ values observed in barley fell within the range of several tabulated values, including FEDNA [16], Rostagno et al. [17], and WPSA [15], but were higher compared to values reported in CVB [18], INRAE [19], and NRC [20]. Exceptionally, the AME_n_ value of B3 (3142 kcal/kg) was higher than all reported values in the previous tables, which is likely a result of its high starch concentration (54.9%) and low CP (8.1%) and total NSP (10.1%) contents compared to barley grains used in other evaluations. AME_n_ values in rye were in agreement with reported values from WPSA [15] and NRC [20] tables but lower compared with values reported in FEDNA [16] and Rostagno et al. [17] and higher than the published value in INRA [19] tables. AME_n_ values observed in wheat were consistent with those reported in several tables, including INRA [19], CVB [18], Rostagno et al. [17], WPSA [15], FEDNA [16], and NRC [20]. The variations in the reported values may be partly attributed to the chemical composition of the ingredient tested, such as differences in CP, starch, and antinutritional factor contents. Additionally, differences in the experimental procedures and the type and age of the birds used in the studies may also account for some of the variability observed [1].

In terms of energy metabolisability, rye exhibits the lowest value compared to barley and wheat. This discrepancy can be attributed to the composition of rye’s cell wall carbohydrates, primarily arabinoxylans, which are present in higher concentrations compared to barley and wheat [21], and to the higher levels of fructans and soluble dietary fibre in rye, which may contribute to increased viscosity and hinder the activity of endogenous peptidases [22]. Additionally, rye exhibited higher values for SC and WRC, which could also be linked to lower nutrient digestibility. In the same context, Antoniou and Marquardt [23] observed that the viscous and sticky properties, and the ability to retain large volumes of water in rye, can be attributed to its high concentration of pentosans, which may interfere with nutrient utilisation. On the other hand, the low nutritional value observed in barley has been associated with β-glucans, the principal endosperm and aleurone cell wall component [24]. Water-soluble fractions of arabinoxylans and β-glucans can comprise up to 30% of total NSP content in cereals [25,26]. These compounds possess high viscosity, leading to reduced digestibility and absorption of all nutrients [27]. The effects tend to be more pronounced in the rye-based diet than in the barley-based diet [28].

**Figure 2:**
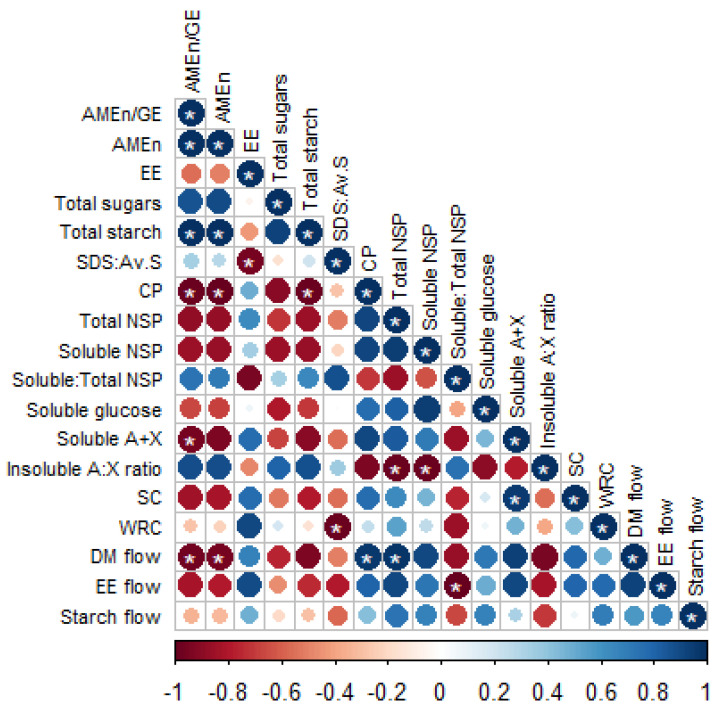
Correlation analysis between in vivo parameters, chemical composition, and physicochemical properties in barley (*n* = 4). The * symbol denotes a statistically significant correlation (*p* < 0.05). The colours scale (Pearson’s r ranging from −1 to +1) indicates whether the correlation is positive (blue) or negative (red). Abbreviations: AME_n_, nitrogen-corrected apparent metabolisable energy; GE, gross energy; EE, ether extract; SDS:Av.S, slowly digestible to available starch ratio; CP, crude protein; NSP, Non-starch polysaccharides; A, arabinose; X, xylose; A:X, arabinose to xylose ratio; SC, swelling capacity; WRC, water retention capacity; and DM, dry matter.

Varietal differences were more pronounced in barley and wheat than in rye. In barley, AME_n_ value varied among different varieties. Similarly, Villamide et al. [29] observed differences in the energy value among eight cultivars of barley when AME_n_ was determined with both broiler chicks and adult roosters. Nutritional variability among varieties or cultivars in barley was reported in many studies [30,31,32]. Among the cereal species, barley has been identified as one of the most variable cereal grains in terms of its energy value [33], and this variability is not reflected in feed tables [34]. Jeroch and Dänicke [35] stated that feeding value in barley is influenced mainly by the content of starch, crude fibre, and total fibre, while Villamide et al. [29] reported no relationship between AME_n_ in barley cultivars and their chemical composition. In the current study, the AME_n_ was correlated positively with starch content (r = 1.00; *p* < 0.001) and the insoluble arabinose to xylose ratio (r = 0.88; *p* < 0.05) and negatively with CP (r = −0.99; *p* < 0.05), total NSP (r = −0.88; *p* = 0.119), and soluble arabinose and xylose (r = −0.93; *p* = 0.069) contents in barley (Figure 2). The variability of energy value in barley based on chemical and physical properties, interaction between the nutrient and antinutrient components, and responses to enzyme supplementation was comprehensively discussed in a recent review by Perera et al. [36].

The AME_n_ value of wheat also varied depending on the variety. Several studies, including those conducted by Del Alamo et al. [37] and Smeets et al. [38], have reported the impact of wheat cultivar on the AME_n_ value. Karunaratne et al. [39] also observed the impact of cultivar on wheat energy value when two wheat cultivars from six different Canadian wheat classes were evaluated, with no correlation found between AME and starch digestibility. A study by Choct et al. [40] also demonstrated a large variation in the AME value of wheat when a total of 81 wheats were assayed for energy value in broilers. Additionally, AME was negatively correlated with all fractions of NSP. In this study, with four wheat varieties, no clear relationship between AME_n_ and the chemical composition of wheat was observed.

### 4.3. Enzyme Addition Response

Broilers fed diets with exogenous enzymes had lower excretion of GE, starch, N, and EE. It has been shown that the enzyme-induced lower viscosity limited microbial proliferation, which otherwise competes for nutrients, thus improving the digestibility of DM, EE, total NSP, and energy [41]. Other authors have also reported a positive response to enzymes on energy digestibility in barley [42], wheat, or rye diets [43]. This also confirms the results of Ravindran et al. [44] showing that the simultaneous inclusion of xylanase and phytase in wheat-based broiler diets was beneficial in terms of protein and energy utilisation.

The ΔAME tended to be greater in the rye-based diet than in the barley- and wheat-based diets, while Marquardt et al. [28] and Lázaro et al. [41] observed that improvement in response to enzyme addition for AME_n_ tended to be greater for barley than for rye or wheat diets. The differences between experiments might be due to different cereal grain composition, levels of inclusion, and number of varieties used, as well as the number, type, and dose of enzymes added. The variety influenced ΔAME in barley, with varieties having low metabolisability showing higher responses to enzyme addition. This variable response to supplemental enzymes could be attributed to variations in barley anti-nutritional composition, mainly β-glucan [31] and starch structure [45,46]. A differential effect of enzyme addition on AME_n_ of barley cultivars was also reported by Rotter et al. [47], who obtained the greatest response to enzymes for barley cultivars with the greatest viscosity, and by Villamide et al. [29], who stated that the AME_n_ of enzyme-supplemented barley can be estimated based on chemical parameters, mainly crude fibre and NSP, when working with eight different cultivars. A previous study has found that the response to enzyme supplementation for both barley and wheat cultivars was dependent on the nutritional value of the cereal grain without supplementation [30]. Ravindran et al. [44] stated that the magnitude of response to added enzyme was influenced by the AME of the wheat, as the improvements were greater in the low-AME wheat than in the normal AME wheat. Similarly, Flores et al. [48] found that the best responses to exogenous xylanases are obtained with wheat of the lowest energy value. In their study, the degree of response to enzyme addition was negatively correlated with the true metabolisable energy values of the diets. In the current study, ΔAME in response to enzyme addition was correlated negatively with AME_n_ content across all cereals. Knowledge of variation in enzyme response among varieties could be used to determine the economic merit of supplementing with enzymes.

### 4.4. Ytterbium as Indigestible Marker

Given the comparable performance of Yb_2_O_3_ to TiO_2_, as evidenced by the regression analysis in this research, Yb_2_O_3_ presents a promising substitute that may circumvent potential regulatory or safety issues associated with TiO_2_. The lack of significant interaction between marker type and cereal species, varieties, or enzyme supplementation is important as it underscores the robustness of these markers in varied dietary conditions. Our study’s use of Yb_2_O_3_ as a digestibility marker aligns with findings across various species, demonstrating its effectiveness. Teeter et al. [49] showed that Yb-labelled soybean meal exhibits similar flow rates to ferric oxide in the digestive tract of broiler chicks. Similarly, Deering et al. [50] found that ytterbium acetate allowed for similar protein digestibility estimates compared to acid-insoluble ash and chromic oxide in leader prawns. Additionally, Delagarde et al. [51] confirmed that Yb_2_O_3_ provides accuracy comparable to chromic oxide in estimating faecal dry matter output in dairy cows. The inclusion of Yb_2_O_3_ at a minimal concentration of 50 mg/kg in animal feed might be both cost-effective and safer for digestibility studies. In terms of its usage, Yb_2_O_3_ (CAS number 1314-37-0) is not classified as a food additive in Europe. It is not a hazardous substance or mixture according to Regulation (EC) No. 1272/2008 [52].

## 5. Conclusions

The evaluation method used in this study highlighted the large degree of variability among and within cereals. Both cereal species and variety were important for determining the metabolisability of energy and the response to feed enzymes, which is not reflected in the tabulated values or the NIR estimates. Focusing on the differences between the nutritional value of ingredients will allow for the evaluation and ranking of a large number of ingredients regardless of experimental conditions. Correlations within each ingredient may provide a potential tool for classifying probable factors that impact the cereal grains’ feeding value. Moreover, ytterbium oxide appears to be a promising marker for digestibility studies.

## Figures and Tables

**Table 1:** Composition of the adaptation and experimental diets (%, as-fed basis).

Item	Adaptation Diet (d1–15)	Experimental Diets ^4^ (d16–25)
*Ingredients*		
Corn	32.28	32.28
Wheat	40	-
Test ingredient ^1^	-	40
Extruded soybean	18	18
Processed animal protein ^2^	8	8
L-Lysine	0.34	0.34
DL-Methionine	0.31	0.31
L-Threonine	0.19	0.19
Isoleucine	0.125	0.125
Tryptophan	0.02	0.02
Salt	0.33	0.33
Vitamin and mineral premix ^3^	0.4	0.4
*Calculated composition*		
AME (kcal/kg)	3246	
Crude protein	19.6	
Calcium	0.48	
Phosphorus	0.5	

^1^ Each one of the twelve cereals evaluated. ^2^ Derived from the processing of poultry products, 65% crude protein. ^3^ Provided per kg of feed: vitamin A (retinol acetate) 10,000 IU; vitamin D (vitamin D3) (cholecalciferol) 539 4800 UI; vitamin E/tocopherol) 45 mg; vitamin K3 (MNB, menadione nicotinamide bisulfate) 3 mg; vitamin B1 (tiamin mononitrate) 3 mg; 540 vitamin B2 (riboflavin) 9 mg; vitamin B6 (piridoxin chlorohydrate) 4.5 mg; vitamin B12 (cyanocobalamine) 0.04 mg; nicotinamide 51 mg; 541 pantothenic acid (calcium D-pantothenate) 16.5 mg; biotin (D−(+) biotin) 0.15 mg; folic acid 1.8 mg; choline chloride 350 mg; iron (iron 542 sulphate monohydrate) 54 mg; zinc (Zn, zinc oxide) 66 mg; manganese (Mn, manganese oxide) 90 mg; iodine (I, calcium iodine anhydrate) 543 1.2 mg; selenium (Se, sodium selenate) 0.18 mg; copper (Cu, copper sulphate pentahydrate) 12 mg. ^4^ Titanium dioxide (0.2%) and ytterbium oxide (0.005%) were added as indigestible markers. Enzyme inclusion (in enzyme-supplemented diets): phytase (1000 FTU/kg), xylanase (16,000 BXU/kg), and β-glucanase (20,000 BU/kg). All enzymes were sourced from AB Vista (Marlborough, UK).

**Table 2:** Analysed nutrient concentrations of 4 different varieties of barley, rye, and wheat (% as-fed basis unless otherwise stated).

	Barley				Rye				Wheat			
Item (%)	B1	B2	B3	B4	R1	R2	R3	R4	W1	W2	W3	W4
Dry matter	88.6	88.2	88.4	88.4	87.7	88.3	88.6	88.2	87.4	87.6	88.3	87.3
Ether extract	1.65	1.40	1.01	1.13	0.87	0.89	0.86	0.94	1.14	1.13	1.15	0.90
Crude protein	9.6	9.09	8.15	9.87	8.7	6.22	6.68	8.64	9.63	10.9	13.7	10.6
Gross energy (kcal/kg)	3870	3850	3827	3839	3745	3763	3786	3796	3786	3800	3880	3787
Total sugars	2.2	2.5	2.9	1.4	4.8	6.0	5.2	6.0	2.7	3.6	2.3	1.6
Calcium	0.04	0.05	0.04	0.03	0.03	0.03	0.03	0.04	0.04	0.04	0.05	0.03
Phosphorus	0.32	0.29	0.27	0.29	0.31	0.29	0.26	0.30	0.35	0.32	0.32	0.29
Zinc (mg/kg)	47	45	21	28	20	26	24	21	18	28	26	23
Amino acids												
Aspartic acid	0.60	0.61	0.58	0.64	0.58	0.53	0.53	0.59	0.50	0.55	0.64	0.52
Glutamic acid	2.27	2.00	1.85	2.33	1.95	1.14	1.27	1.91	2.77	3.08	4.15	3.03
Serine	0.47	0.44	0.40	0.46	0.42	0.29	0.31	0.42	0.46	0.48	0.61	0.49
Histidine	0.24	0.20	0.19	0.21	0.19	0.15	0.15	0.20	0.22	0.24	0.31	0.18
Glycine	0.45	0.38	0.32	0.36	0.36	0.25	0.28	0.37	0.37	0.42	0.50	0.45
Threonine	0.38	0.36	0.33	0.37	0.32	0.26	0.26	0.34	0.33	0.31	0.39	0.30
Arginine	0.53	0.5	0.46	0.51	0.47	0.39	0.39	0.49	0.50	0.57	0.65	0.53
Alanine	0.41	0.40	0.38	0.41	0.38	0.31	0.31	0.39	0.37	0.39	0.43	0.38
Tyrosine	0.34	0.27	0.27	0.32	0.23	0.18	0.18	0.24	0.30	0.30	0.40	0.32
Valine	0.52	0.42	0.41	0.46	0.38	0.30	0.30	0.38	0.39	0.43	0.49	0.41
Methionine	0.16	0.14	0.13	0.16	0.13	0.09	0.09	0.12	0.14	0.15	0.19	0.16
Phenylalanine	0.49	0.46	0.44	0.53	0.41	0.28	0.29	0.41	0.44	0.52	0.69	0.48
Isoleucine	0.35	0.31	0.30	0.34	0.29	0.22	0.22	0.29	0.32	0.36	0.47	0.34
Leucine	0.62	0.63	0.60	0.62	0.55	0.40	0.41	0.56	0.58	0.67	0.88	0.65
Lysine	0.36	0.35	0.32	0.37	0.32	0.23	0.26	0.34	0.29	0.35	0.41	0.30
Proline	1.01	0.87	0.81	1.03	0.78	0.46	0.49	0.77	0.90	1.01	1.44	0.99
Swelling capacity	313	287	280	297	448	499	477	397	184	181	170	182
Water retention capacity	107	107	103	103	119	128	128	123	81	75	80	80
NIR predictions *												
AME_n_, %GE	72.5	72.3	75.0	71.7	67.5	68.3	69.2	67.6	80.7	80.4	79.1	80.6
AME_s_, %GE	74.1	73.9	76.4	73.4	69.0	69.4	70.3	69.0	82.4	82.2	81.5	82.4

Abbreviations: NIR, near-infrared reflectance technology; AME_n_, nitrogen-corrected apparent metabolisable energy; AME_s_, apparent metabolisable energy standardised for retained nitrogen equal to 50% of nitrogen intake; GE, gross energy. * The AME_n_ values were estimated using NIR calibrations provided by Evonik Operations GmbH (Essen, Germany), derived from wet-chemistry-analysed parameters and the WPSA [15] equation for AME_n_. Calibration accuracy was assessed using a standard error of cross-validation (SECV), with values of ±0.082 MJ AME_n_/kg for barley and ±0.154 MJ AME_n_/kg for wheat and rye. Gross energy (GE) was estimated with errors of ±0.039 MJ GE/kg for barley, ±0.037 MJ GE/kg for rye, and ±0.036 MJ GE/kg for wheat. Relative errors for AME_n_/GE were ±0.47 for barley and ±0.86 for wheat and rye.

**Table 3:** Analysed starch fractions and non-starch polysaccharides (NSP) constituent sugars of 4 different varieties of barley, rye, and wheat (as-fed basis).

	Barley				Rye				Wheat			
Item	B1	B2	B3	B4	R1	R2	R3	R4	W1	W2	W3	W4
Starch fractions ^1^												
Rapidly digestible starch	22.5	22.3	19.8	18.3	29.4	29.4	28.3	25.3	25.6	22.8	22.7	27.7
Slowly digestible starch	28.9	30.4	34.6	32.3	26.7	28.2	28.4	27.1	34.2	35.6	34.2	34.0
Available starch	51.3	52.6	54.4	50.6	56.2	57.6	56.7	52.4	59.8	58.4	56.9	61.6
Resistant starch	0.5	0.8	0.5	0.7	0.3	0.4	0.3	0.4	0.4	0.7	0.3	0.3
Total starch	51.9	53.4	54.9	51.3	56.5	58.0	57.0	52.8	60.2	59.2	57.2	61.9
Soluble NSP (g/100 g)												
Rhamnose	0.0	0.0	0.0	0.0	0.0	0.0	0.0	0.0	0.0	0.0	0.1	0.0
Fucose	0.0	0.0	0.0	0.0	0.0	0.0	0.0	0.0	0.0	0.0	0.0	0.0
Arabinose	0.5	0.4	0.3	0.5	1.1	1.1	1.1	1.3	0.6	0.5	0.5	0.6
Xylose	0.7	0.5	0.4	0.6	1.9	1.9	1.8	2.3	1.1	0.8	0.8	1.0
Mannose	0.2	0.2	0.1	0.2	0.1	0.2	0.2	0.2	0.1	0.1	0.1	0.1
Galactose	0.2	0.2	0.2	0.2	0.2	0.2	0.2	0.2	0.3	0.3	0.3	0.2
Glucose	3.6	4.1	3.2	4.4	1.3	1.2	1.3	1.4	0.5	0.5	0.3	0.4
Glucuronic acid	0.0	0.0	0.0	0.0	0.0	0.0	0.0	0.0	0.0	0.0	0.0	0.0
Galacturonic acid	0.0	0.0	0.0	0.0	0.0	0.0	0.0	0.0	0.0	0.0	0.0	0.0
Insoluble NSP (g/100 g)												
Rhamnose	0.0	0.0	0.1	0.1	0.0	0.0	0.0	0.0	0.1	0.0	0.1	0.0
Fucose	0.0	0.0	0.0	0.0	0.0	0.0	0.0	0.0	0.0	0.0	0.0	0.0
Arabinose	1.7	1.6	1.3	1.5	1.4	1.4	1.4	1.5	1.4	1.2	1.4	1.0
Xylose	3.7	3.8	2.1	3.7	2.3	2.2	2.3	2.5	3.4	2.0	2.3	1.8
Mannose	0.3	0.3	0.2	0.3	0.3	0.4	0.4	0.4	0.3	0.2	0.2	0.2
Galactose	0.2	0.2	0.1	0.2	0.2	0.2	0.3	0.2	0.2	0.1	0.2	0.1
Glucose	3.9	3.8	2.1	3.9	2.3	2.5	2.5	2.4	4.0	2.0	2.0	1.4
Glucuronic acid	0.0	0.0	0.0	0.0	0.0	0.0	0.0	0.0	0.0	0.0	0.0	0.0
Galacturonic acid	0.0	0.0	0.0	0.0	0.0	0.0	0.0	0.0	0.0	0.0	0.0	0.0
Total NSP (g/100 g)	15.1	15.1	10.1	15.4	11.1	11.5	11.5	12.5	11.9	7.8	8.2	6.8
% Soluble NSP	34.5	36.1	41.5	38.1	41.5	40.6	39.7	44.0	21.7	28.6	24.9	33.8
% Insoluble NSP	65.5	63.9	58.5	61.9	58.5	59.4	60.3	56.0	78.3	71.4	75.1	66.2

^1^ g/100 g polysaccharides (×0.9 factor), determined according to Englyst et al. [8].

**Table 4:** Analysed activity of phytase, xylanase, and β-glucanase of experimental diet samples.

				Enzyme Activity		
				Phytase	Xylanase	β-Glucanase
Diets	Cereal	Enzymes ^1^	Variety ^2^	(FTU/kg)	(BXU/kg)	(BU/kg)
B1	Barley	−	B1	<50	<2000	8810
B2			B2	<50	<2000	10,800
B3			B3	<50	<2000	6720
B4			B4	<50	<2000	7340
B1+		+	B1	1590	12,800	32,000
B2+			B2	1560	15,600	30,500
B3+			B3	1370	14,300	32,000
B4+			B4	1390	17,600	31,900
R1	Rye	−	R1	<50	<2000	11,500
R2			R2	<50	<2000	13,800
R3			R3	<50	<2000	17,600
R4			R4	<50	<2000	10,600
R1+		+	R1	1900	17,100	39,600
R2+			R2	1590	17,300	28,700
R3+			R3	1490	17,200	29,600
R4+			R4	1620	16,800	31,900
W1	Wheat	−	W1	<50	<2000	7420
W2			W2	<50	<2000	6490
W3			W3	<50	<2000	9120
W4			W4	<50	<2000	7440
W1+		+	W1	1140	14,000	30,300
W2+			W2	1640	17,400	29,900
W3+			W3	1550	15,500	32,200
W4+			W4	1110	16,500	24,700

^1^ Without (−) or with (+) supplementation of 1000 FTU/kg, 16,000 BXU/kg, and 20,000 BU/kg of phytase, xylanase, and β-glucanase, respectively. ^2^ Four different varieties of each cereal species.

**Table 5:** Influence of cereal species inclusion, enzyme supplementation, and the marker type on the flow of gross energy (GE), starch, nitrogen (N), and ether extract (EE) in excreta (/kg dry matter intake) in growing broiler diets.

		GE (kcal)	Starch (g)	N (g)	EE (g)
*Main effects*					
Cereal (C) ^1^	Barley	1191 ^b^	15.68 ^c^	11.63	20.97 ^b^
	Rye	1287 ^a^	21.08 ^a^	11.98	23.32 ^a^
	Wheat	1093 ^c^	19.36 ^b^	12.01	20.58 ^b^
	SEM ^2^	34.8	0.760	0.245	1.128
Enzymes (E) ^3^	−	1224	19.33	12.19	22.79
	+	1155	18.08	11.56	20.46
	SEM	33.4	0.673	0.231	1.013
Marker (M)	TiO_2_	1197	18.93	11.96	21.57
	Yb_2_O_3_	1181	18.64	11.78	21.26
	SEM	32.1	0.709	0.270	0.987
*p-Values*					
C		<0.001	<0.001	0.124	0.008
E		<0.001	<0.001	<0.001	<0.001
M		0.118	0.490	0.181	0.554
C × E		0.222	0.661	0.303	0.227

^1^ Included at 40% in a basal diet. ^2^ Standard error of the mean. ^3^ Without (−) or with (+) supplementation of 1000 FTU/kg, 16,000 BXU/kg, and 20,000 BU/kg of phytase, xylanase, and β-glucanase, respectively. ^a–c^ Means in the same column within a main effect lacking a common superscript differ (*p* < 0.05). Other interactions between main effects were not included because they were not significant. *n* = 96 for cereal species, *n* = 144 for enzyme supplementation, and *n* = 144 for marker type. These values reflect the total observations per level, considering the two runs and inclusion of both markers.

**Table 6:** Influence of cereal variety inclusion, enzyme supplementation, and the marker type on the flow of gross energy (GE), starch, nitrogen (N), and ether extract (EE) in excreta (/kg dry matter intake) in growing broilers diets.

		Barley-Based				Rye-Based				Wheat-Based			
		GE (kcal)	Starch (g)	N (g)	EE (g)	GE (kcal)	Starch (g)	N (g)	EE (g)	GE (kcal)	Starch (g)	N (g)	EE (g)
*Main effects*													
Variety (V) ^1^	1	1224	15.62	12.06	22.16	1272	20.70	12.15	22.23	1163 ^a^	21.73 ^a^	12.08	22.06
	2	1202	16.53	11.66	21.50	1298	21.71	11.89	24.30	1091 ^b^	19.21 ^ab^	11.97	20.55
	3	1110	14.99	10.79	19.27	1268	21.52	11.79	23.61	1073 ^bc^	18.49 ^b^	12.38	18.08
	4	1228	15.68	11.99	21.04	1308	20.50	12.11	23.15	1025 ^c^	17.76 ^b^	11.48	21.59
	SEM ^2^	38.9	1.289	0.291	1.756	31.1	1.049	0.270	1.119	44.8	1.054	0.625	1.757
Enzymes (E) ^3^	−	1218	16.21	11.84	21.64	1333	21.82	12.26	24.54	1114	19.97	12.81	22.20
	+	1164	15.15	11.41	20.35	1240	20.35	11.71	22.11	1062	18.75	11.56	18.94
	SEM	35.4	1.055	0.191	1.622	25.6	0.741	0.205	0.790	41.3	0.605	0.553	0.932
Marker (M)	TiO_2_	1198	15.76	11.67	20.85	1290	21.36	12.01	23.38	1102	19.57	12.15	20.56
	Yb_2_O_3_	1185	15.59	11.54	20.65	1283	21.23	11.95	23.27	1073	19.05	11.82	19.99
	SEM	35.5	0.984	0.152	1.700	24.7	0.874	0.190	0.678	41.2	0.528	0.533	1.099
*p-Values*													
V		<0.001	0.653	0.004	0.124	0.497	0.770	0.575	0.682	<0.001	0.049	0.390	0.301
E		<0.001	0.009	0.006	0.985	<0.001	0.031	<0.001	0.003	0.002	0.047	<0.001	<0.001
M		0.324	0.778	0.491	0.750	0.699	0.874	0.695	0.911	0.107	0.455	0.222	0.577
V × E		<0.001	0.002	<0.001	0.140	0.206	0.915	0.402	0.209	0.058	0.734	0.125	0.149

^1^ (1–4) Four different varieties of each cereal included at 40% in a basal diet. ^2^ Standard error of the mean. ^3^ Without (−) or with (+) supplementation of 1000 FTU/kg, 16,000 BXU/kg, and 20,000 BU/kg of phytase, xylanase, and β-glucanase, respectively. ^a–c^ Means in the same column within a main effect lacking a common superscript differ (*p* < 0.05). Superscripts for main effects are omitted when the interaction effect is significant. Other interactions between main effects were not included because they were not significant. *n* = 24 for cereal variety, *n* = 48 for enzyme supplementation, and *n* = 48 for marker type. These values reflect the total observations per level, considering the two runs and inclusion of both markers.

**Table 7:** Influence of cereal species and variety on the metabolisability of energy, the nitrogen-corrected apparent metabolisable energy, and the standardised apparent metabolisable energy in cereals and on the increment of energy in enzyme-supplemented diets fed to growing broilers.

Cereal	Variety ^1^	GE (kcal/kg)	Metabolisability_n_^2^ (% GE)	AME_n_ (kcal/kg)	Metabolisability_s_^3^ (% GE)	AME_s_ (kcal/kg)	ΔAME_n_^4^ (kcal/kg diet)
Barley	B1	3870	72.7 ^c^	2814 ^c^	74.5 ^c^	2883 ^c^	86.9 ^a^
	B2	3850	77.2 ^b^	2972 ^b^	78.9 ^b^	3036 ^b^	−13.2 ^b^
	B3	3827	82.1 ^a^	3142 ^a^	83.1 ^a^	3181 ^a^	27.2 ^ab^
	B4	3839	72.4 ^c^	2780 ^c^	74.3 ^c^	2854 ^c^	79.2 ^a^
	SEM		1.55	59.5	1.55	59.5	63.24
	*p*-Value		<0.001	<0.001	<0.001	<0.001	0.008
Rye	R1	3745	67.8	2540	69.5	2605	127.8
	R2	3763	69.3	2608	70.8	2663	57.5
	R3	3786	70.7	2677	72.2	2732	69.2
	R4	3796	68.6	2603	70.0	2658	72.5
	SEM		2.06	77.4	2.06	77.4	47.04
	*p*-Value		0.540	0.371	0.605	0.441	0.362
Wheat	W1	3786	77.2 ^b^	2921 ^c^	78.8 ^b^	2981 ^c^	68.9
	W2	3800	80.4 ^a^^b^	3055 ^bc^	82.2 ^a^^b^	3124 ^b^^c^	83.2
	W3	3880	84.1 ^a^	3261 ^a^	86.4 ^a^	3351 ^a^	−13.2
	W4	3787	84.5 ^a^	3202 ^ab^	86.5 ^a^	3277 ^a^^b^	23.7
	SEM		2.68	102.1	2.68	102.1	67.32
	*p*-Value		0.002	<0.001	<0.001	<0.001	0.123
Barley			76.2 ^b^	2930 ^b^	77.8 ^b^	2991 ^b^	44.1
Rye			69.1 ^c^	2607 ^c^	70.6 ^c^	2665 ^c^	81.8
Wheat			81.3 ^a^	3104 ^a^	83.3 ^a^	3177 ^a^	41.6
SEM			0.79	30.6	0.79	30.7	54.42
*p*-Value			<0.001	<0.001	<0.001	<0.001	0.091

Abbreviations: GE, gross energy; AME_n_, nitrogen-corrected apparent metabolisable energy; AME_s_, apparent metabolisable energy standardised for retained nitrogen equal to 50% of nitrogen intake; SEM, standard error of the mean. ^1^ (1–4) Four different varieties of each cereal. ^2^ Metabolisability of energy to calculate AME_n_. ^3^ Metabolisability of energy to calculate AME_s_. ^4^ Increment of AME_n_ in enzyme-supplemented diets with 1000 FTU/kg, 16,000 BXU/kg, and 20,000 BU/kg of phytase, xylanase, and β-glucanase, respectively. ^a–c^ Means in the same column within a main effect lacking a common superscript differ (*p* < 0.05). *n* = 12 for cereal variety and *n* = 24 for marker type in the comparison of varieties within each cereal species, and *n* = 48 for cereal species and *n* = 72 for marker type in the cereal species comparison. Means for marker type are not presented to avoid overloading the table, as no significant differences were observed between TiO_2_ and Yb_2_O_3_ in any of the analyses.

## Data Availability

The data presented in this study are available upon request from the corresponding author.

## Appendix A

**Table A1:** Variability in composition and energy content of barley, rye, and wheat in different feed tables (/kg as fed).

Cereal	Annotation	CP	Starch	CF	NDF	ADF	EE	Ca	P	Zn	GE	AME_n_	Institution
		(%)								(mg)	(kcal)		
Barley	*Barley*	10.8	52.1	4.25	16.2	5.31	1.7	0.05	0.35	29	3830	2701	Brazilian tables [17]
	*Barley*	9.9	52.3	4.7	18.7	5.6	1.6	0.07	0.34	30	3820	2600	INRAE [19]
	*Barley*	10.2	51.7	4.7	14.1	5.6	1.8	0.05	0.32	30		2552	CVB [18]
	*Barley*	11		5.5			1.8	0.03	0.36	30		2640	NRC [20]
	*Barley 1 (6 Row)*	9.6	50.6	5.6			1.7					2703	WPSA [15]
	*Barley 2 (2 Row)*	11.7	52.2	4.3			2.2					2817	WPSA [15]
	*Barley 1*	11.3	51.9	4.7	18.1	5.5	1.7	0.06	0.32	30		2785	FEDNA [16]
	*Barley 2*	9.6	52.5	4.7	18.1	5.5	1.7	0.06	0.32	30		2750	FEDNA [16]
Rye	*Rye*	10.3	59.6	1.9	14.1	3.1	1.6	1.8	0.1		3749	2870	Brazilian tables [17]
	*Rye*	8.5	53.7	2	12.9	2.8	1.2	0.06	0.3	22	3720	2330	INRAE [19]
	*Rye*	9.3	54	2.1	9.8	3	1.3	0.04	0.31	33			CVB [18]
	*Rye*	12.1		2.2			1.5	0.06	0.46	31		2626	NRC [20]
	*Rye*	10	56.9	2.6			1.7					2495	WPSA [15]
	*Rye*	9.4	55.3	2.3	13.6	3.4	1.3	0.04	0.3	25		2730	FEDNA [16]
	*Rye (German)*	10.1	53.7	2.2	11.2	3.2	1.8	0.03	0.32	25		2750	FEDNA [16]
Wheat	*Wheat*	11.5	56.7	2.37	11.32	3.09	1.61	0.06	0.32	53.11	3810	3039	Brazilian tables [17]
	*Wheat*	11	60	2.4	12.8	3.3	1.4	0.06	0.31	26	3780	2860	INRAE [19]
	*Wheat*	11	59.2	2.3	11.7	3.5	1.5	0.03	0.3	28		2868	CVB [18]
	*Wheat*	11.5		3			2.5	0.05	0.31	28		3120	NRC [20]
	*Wheat*	11.3	61.8	2.6			2.2					3067	WPSA [15]
	*Wheat 1*	12.9	60.1	2.4	10.8	3.2	1.4	0.05	0.29	50		3155	FEDNA [16]
	*Wheat 2*	11.2	60.4	2.4	10.6	3.1	1.4	0.05	0.29	50		3100	FEDNA [16]
	*Wheat 3*	10.2	60.6	2.4	10.3	3	1.4	0.05	0.29	50		3075	FEDNA [16]
	*Wheat (English)*	11	60	2	9.3	3.1	1.6	0.04	0.3	30		3090	FEDNA [16]

Abbreviations: CP, crude protein; CF, crude fibre; NDF, neutral detergent fibre; ADF, acid detergent fibre; EE, ether extract; GE, gross energy; and AME_n_, nitrogen-corrected metabolisable energy.
